# Private Practice—Does It Have a Place?

**Published:** 1991-09

**Authors:** Ian S. Bailey

**Affiliations:** Consultant Physician, Medical Advisor to Western Provident Association


### Private Practice — Does it have a place?

**Based on a talk at the Medical Education Centre, Southmead Hospital, Bristol 24.1.1991**

**Ian S. Bailey, MD, FRCP**
Consultant Physician,
Medical Advisor to Western Provident Association

The National Health Service and the private sector should be complementary; each needs the other. It is highly improbable that the Health Service will be privatised; much more likely that private practice will be publicised by bringing it within the National Health Service.

This article discusses the size and growth of the private sector, looks at cost containment and quality and examines the problems and opportunities of the new Health Service structure.

In 1979 the Royal Commission on the National Health Service reported: ''We have reached no conclusion about the overall balance of advantage or disadvantage to the National Health Service of the existence of a private sector. Whichever way it lies, it is small''. In that year the pay beds in NHS hospitals were markedly reduced. At that time over half of all NHS Consultants were working full-time; maximum part-time Consultants, on average, derived one-third of their income from private practice; about half the patients receiving treatment in private beds were covered by health insurance. People chose private treatment for privacy, for the convenience of booking a date for admission, to guarantee seeing a particular Consultant and to reduce the waiting time for an outpatient appointment or inpatient treatment. The Royal Commission noted that pay beds in the United Kingdom had decreased from just over 6,000 in 1965 to just under 3,000 in 1979 and that patients treated in pay beds had declined from just over 120,000 in 1972 to just under 94,000 in 1977.

Private beds in NHS hospitals have remained at around 3,000 but the occupancy has been low and the revenue has remained steady between £50 and £60 million per annum but since 1988 has increased to just over £90 million per annum. There are now 18 private units on NHS sites and it is possible that private beds in NHS hospitals will outstrip provision in the private sector within 5 years. Private beds outside the NHS have increased from just over 6,500 in 1979 to 10,300 in 1988 and now to 11,000. The average occupancy is around 60%; some 80% of patients in private beds are now fully insured. Commercial sector beds have increased threefold; the number of charitable beds is unchanged. 25 new hospitals were opened in the 4 years between 1986 and 1989. More private hospitals have intensive care facilities allowing complex surgery. The commonest procedures remain wisdom teeth, endoscopy, D&C and excision of skin lesions.[1]

Between 6 and 7 million people are covered by health insurance, a three-fold increase since 1979. Premiums are now approximately £1 billion. Some people pay for their own private treatment and payment for private provision is now about £1.25 billion. For most of England there are between 15 and 20 private beds for 100,000 population but in the four London Districts there are between 30 and 40 per 1,000 compared with the NHS provision of between 200 and 300 for 100,000 population. The majority of procedures in the private sector are simple, but an increasing number of complex operations are now being performed. About two-thirds of the income to the private sector goes to hospitals and about one-third to professional fees. Professional fees have increased broadly in line with inflation, but the cost of hospitals has increased at a greater rate than inflation, and there has been a particularly marked increase in theatre fees and the cost of drugs, dressings and consumables. Hospital accommodation charges are frequently above £250 a day and can reach £1,000 if intensive care is needed. The additional cost from theatre fees, drugs, dressings and consumables can double the daily rate.

There has been increasing interest in standards in private practice. The Conference of Medical Royal Colleges has recommended that health insurers should give specialist recognition to those who hold substantive NHS Consultant posts or who are accredited by the appropriate Royal College and have suggested that private hospitals should use the same criteria in determining admitting rights. The General Medical Council is, during 1991, establishing an Indicative Specialist Register on similar criteria, and from 1st January 1992 will publish an indicator ''T'' of specialist training and of the speciality; Surgery, Obstetrics and Gynaecology, Ophthalmology, Anaesthetics, Medicine, Psychiatry, Pathology, Clinical Radiology, Clinical Oncology, Occupational Medicine, Public Health Medicine and General Practice. Admitting rights to some hospitals cease at the age of 70, and some health insurers may deny benefits for a consultation or treatment by specialists over the age of 70.

There is greater understanding that private costs need to be contained if insurance and private practice is to continue. Hospital accommodation costs have to some extent been controlled by negotiation; other costs have not been contained. There has recently been a greater public awareness of such costs and more and more people are looking at and questioning their hospital bill in an endeavour to reduce hospital costs without any fall in quality. The Western Provident Association has produced ten rules to beat medical inflation. Subscribers are asked to think first of NHS treatment, to remember that it is their money through subscriptions which is being spent, to compare charges between local hospitals and to ask about extras and mark-ups. Subscribers should look at the bill and check that this is correct; they should be certain that the treatment is really necessary and, if in doubt, discuss this with their General Practitioner or if necessary, seek a second opinion.

Medical costs do not present so serious a problem, but there are anomalies. Procedures have always been rewarded more than consultation and opinion; specialities other than surgery are becoming involved in procedures; the reward in private practice can, therefore, vary greatly between specialities unlike the Health Service where, apart from merit awards, all Consultants receive the same salary. The British Medical Association Schedule of Fees, produced in 1989 and updated annually, was derived from a large data base in the private sector in the UK but did take into account international comparison, with adjustments to conform with UK practice. The Guidelines are almost entirely concerned with procedures. The 1990 Schedule upgraded relative values by 9.5% to allow for inflation, and on the 1st April 1991 relative values were increased by a further 10.5%, and some procedures have been revalued on professional advice. New procedures, for example Laparoscopic Cholecystectomy, have been given a value slightly greater than conventional Cholecystectomy. The Western Provident Association has accepted the British Medical Association Schedule as a guide to maximum benefit but does recognise that there are certain distortions. A consultation fee which relates to time might favour Psychiatrists and retired Physicians! The recommended fee for procedures is perhaps too high.

Gastrointestinal endoscopy carries a recommended fee some 10 times that which will be paid in the Health Service to a Consultant for the same procedure. Public pressure from individuals and companies concerned to reduce costs of health insurance, and external competition and package deals, and from practice elsewhere in Europe may exert a downward pressure on these recommended fees. Professional Societies and Royal Colleges are likely to be involved increasingly in standards of competence for those performing procedures, professional guidelines on whether and when procedures might be necessary and in advising appropriate fees.

"Epidaurus" has recently been introduced by the Western Provident Association and Mondial Assistance.[2] Uninsured people on Health Service waiting lists for a number of conditions, who wish to pay for their treatment, will be given a quotation for a local private hospital or Health Service pay bed, a more distant hospital in UK and a hospital elsewhere in Europe, chosen in collaboration with Mondial Assistance and known to be of international standing. The patient will make the choice of where they would prefer the operation to be performed. For many procedures the cost in France is less than that in the United Kingdom. A Mori survey has shown that 30% of people would be prepared to travel if the cost was significantly lower. The procedures are as follows:

| | |
|---|---|
| Cataract | Wisdom Teeth |
| Tonsils & adenoids | Varicose veins |
| Inguinal hernia repair | Cholecystectomy |
| Haemorrhoidectomy | Hysterectomy |
| Laparoscopic sterilisation | Transurethral prostatectomy |
| Vasectomy | Hip replacement |
| Knee replacement | Meniscectomy |
| Hallux Valgus | Dupuytren's Contracture |
| Carpal Tunnel Syndrome | |

None is of such complexity that complication would be frequent. All patients will be accepted on a Medical Declaration from the doctor and the patient, which gives details of previous illness, drug treatment, and tells "Epidaurus" that there has already been specialist referral. The General Practitioner will be asked to state that he has no objection to the treatment proposed, and will agree to resume treatment when the patient returns home. The treating hospital will inform not only the General Practitioner but also the specialist on whose list the patient has been placed. Short term complications will be covered within the package deal by the treating hospital. Any serious deterioration of health, requiring prolonged treatment or repatriation will be covered by insurance and administered by Mondial Assistance. It is too soon to say how frequently this will be taken up. It is possible the E112 arrangement, which allows Purchasing Health Authorities and, in theory though not yet in practice, Fund Holding practices to pay for treatment elsewhere in Europe might be used, either directly or through "Epidaurus". It is possible the list could be extended. It should work well for the patient; it may allow a downward pressure on medical costs in the UK by providing an external market.

Doctors and patients do not always understand the limitations of health insurance.[3] In individual membership any condition present at the time of registration must, on good insurance principle, be excluded. People often think that a condition not declared can then be subject to benefit. The general insurance rule is that if a condition was known to the subscriber, benefit cannot be given unless there is clear evidence the condition had taken a different turn since registration. There are particular difficulties with varicose veins, and claims within a short time of registration suggest that these are the result of insurance, rather than of a new clinical need. A medical report from the General Practitioner or specialist will usually establish whether there is such need.

Most private health insurance will exclude maternity care, in vitro fertilisation and its complications and consequences; drug and alcohol abuse; cosmetic surgery; transplant surgery; chronic dialysis; vasectomy and sterilisation; sexually transmitted diseases; HIV and AIDS; self inflicted injuries and injuries arising from professional sport and some particularly dangerous sports; long term psychiatric treatment; convalescence and nursing home care; routine medical examinations; preventive medicine; primary care by the General Practitioner and most complementary medicine. Prolonged physiotherapy, speech therapy and psychological support would be looked at critically; rehabilitation would be covered if there was a supporting clinical report. There is variation between insurers. All subscribers should be encouraged to read the rules and to weigh cost against benefit. The purpose of health insurance is to cover acute illness on a short term basis; most people seem to expect cover for long term and chronic conditions, and this clearly cannot be given in the interest of the generality of subscribers, and of an acceptable subscription rate.

Hormone replacement treatment is a particular example. A good case can be made for oral replacement by oestrogen, or oestrogen and progestogen if there is an intact uterus, to improve well-being, to diminish vascular risk to heart and brain and to diminish the risk of osteoporosis and fracture. Some have argued that this should be offered to all women after the menopause; the net cost of treatment from 50 to 65, taking into account the saving from fewer fractures and less vascular disease has been calculated at £137 for unopposed oestrogen therapy and £521 for combined therapy.[4] In the last few years there has been a considerable increase in claims for hormone replacement treatment by implantation, at a cost to each patient of around £500 a year. There is some evidence that such treatment improves spinal bone mass but no clear evidence yet on the relative long term benefit compared with oral treatment. Some women prefer a six monthly implant to oral treatment; a few people cannot take oral or patch treatment. Health insurance cannot cover such long term treatment but would cover the cost of an implant performed at the time of a hysterectomy and ovariectomy.

A more difficult problem is cancer chemotherapy. Health insurance looks sympathetically at such treatment and at terminal nursing care. We do, however, depend upon professional guidelines on effective treatment, and continued discussion on the cost benefit of such treatment. Should adjuvant chemotherapy be given to women with carcinoma of the breast, with no involvement of lymph nodes?[5] Is platinum helpful in advanced ovarian cancer?[6] How important is it to look at the cost and benefit of procedures, and to ask how these relate to other ways in which the money might be spent? (Rationing Surgery, The Times 31.12.1990 "Medicine is about saving lives but it is also about the agony of choice").

General Practitioners should use the NHS when appropriate for insured patients, should make sure that any private referral is to a recognised specialist and the fees are reasonable, as judged by the BMA and other schedules, and that the condition falls within the rules of private insurance. It is helpful when the General Practitioner acts as an advocate for the patient to the insurer, supporting the case by relevant facts. Specialists should, where possible, charge fees within the accepted norms and when in excess of these should make this known to the subscriber and negotiate the excess or write to the insurer to explain why the fee needs to be higher than the scheduled benefit. Like the General Practitioner, the specialist should ensure that the condition falls within the rules and should have regard to cost benefit, professional guidelines and controlled trials.

There are opportunities and dangers to private practice from the present Health Service re-organisation. Contracting out might increase the occupancy of private hospital beds and might, therefore, decrease costs for individual treatment. General Practitioner budgets might diminish the "gatekeeper" function of the General Practitioner, by increasing referrals to the private sector. NHS hospitals, particularly Trusts, may wish to increase private practice and may even allow a differential salary structure for those specialists encouraging new business.

Consultants may be encouraged, and may wish to conduct private practice in their hospitals; there may be fewer Consultants in the independent private hospitals and the possiblity there will be an increase in the small number of Consultants in whole-time private practice. Tax relief on insurance premiums for those over 60 will reduce the cost of the age-related premiums but is likely to have only a marginal effect on the uptake of insurance by elderly people. There will be a greater involvement of patients and the public in decision making, both for their own treatment and in priorities, and this will apply both to the Health Service and to the private sector. If the Health Service improves, the demand for private practice may diminish.

But will the Health Service improve? Some consider that the General Practitioner Contract 1990, the Internal Market of Purchasing Authorities and Providing Hospitals, Trust Hospitals and Budget Holding Practices 1991 will improve Health Services by leading to greater efficiency and to patient and doctor led priorities. Others consider that there is no evidence that the changes will work, that information systems are inadequate, that priorities will be determined by Managers and Accountants, that Budget Holding Practices will be too small to be effective and that there will be unexpected ill effects. All agree that better organised Health Services need greater funding but now accept that however great the funding not all beneficial treatments can be offered to all patients. Many consider that there should be greater funding to reduce the social and economic causes and consequences of ill health. The conflicting statements of politicians cause confusion and dismay to public and health professionals. There would be merit in taking health care out of party politics perhaps by bringing it under control of a Committee of the House of Commons. "The share of national resources which should be devoted to health care and the method of raising resources are primarily matters for political decision, but when it comes to allocation of resources within the established health budget the knowledge and skill of health professionals are essential to informed decision making." [8]

Though there is no evidence that the present re-organisation will work, neither was there any evidence that earlier re-organisations nor even the introduction of the Health Service in 1948 would work. If the present changes are to be beneficial they need the co-operation and understanding of doctors, other health professionals and the public. There is perhaps a better chance than in earlier re-organisations of success if the present arrangements encourage a critical approach to expenditure on health care; a greater use of controlled trial evidence, clinical protocols, outcome studies and quality assessment; and audit to determine that what is known is being applied. Government must be prepared to make changes where unanticipated ill effects occur. Teaching, training and research are all at risk. We should ask, with T. S. Eliot,

"Where is the wisdom we have lost in knowledge,
Where is the knowledge we have lost in information?"

and remember

"The bitterness of low quality remains long after the sweetness of low price is forgotten," [9]

and with Caius Petronius AD66

"We trained very hard, but it seemed that every time we were beginning to form up into teams we would be re-organised. I was to learn later in life that we tend to meet any new situation by re-organising and a wonderful method it can be for creating an illusion of progress while producing confusion, inefficiency and demoralisation." [10]

### REFERENCES

1. PRIVATE MEDICINE: The NHS's Safety Value. *BMJ* 1991; **302:** 164-165.
2. "EPIDAURUS". *BMJ* 1991; **303:** 807.
3. BAILEY, I.S. Correspondence; *The Consultant.* Autumn 1990.
4. OSTEOPOROSIS. Royal College of Physicians. 1990: 196.
5. HILLNER, B.E., SMITH, T.J. Efficacy & Cost Effectiveness of Adjuvant Chemotherapy in Women with Node Negative Breast Cancer — A Decision Analysis Model. *N. Engl.J. Med. 1991;* **324:** 160-168.
6. CHEMOTHERAPY IN ADVANCED OVARIAN CANCER. *BMJ* 1991; **303:** 884-893.
7. PROFESSOR SIR BRIAN THWAITES. Letter to The Times. 10.1.1991.
8. BLACK, D.A.K. Paying for Health. *J.Med. Ethics,* 1991; **17:** 117-123.
9. BAILEY, I.S. A Clinicians view of Management. Hospital Update. 1986; 85-87.
10. QUOTED BY ALAN MAYNARD. Ronald Grieve Lecture. Expenditure on Health Care. Hong Kong 2.9.1990.

